# Adenoid Cystic Carcinoma of Bartholin’s Gland Clinically Mimics Endometriosis, A Case Report

**Published:** 2014-11

**Authors:** Mojgan Akbarzadeh-Jahromi, Fatemeh Sari Aslani, Navid Omidifar, Sedigheh Amooee

**Affiliations:** 1 Department of Pathology, School of Medicine, Shiraz University of Medical Sciences, Shiraz, Iran;; 2 Department of Gynecology and Obstetrics, School of Medicine, Shiraz University of Medical Sciences, Shiraz, Iran

**Keywords:** Bartholin’s gland, Adenoid cystic carcinoma, Pain

## Abstract

Adenoid cystic carcinoma of Bartholin’s gland is a rare malignant tumor of female genital tract. We report a case of a 42-year-old woman, presenting a palpable painful mass and burning sensation on the left side of vulva during the preceding two months. Based on examination, a solid fixed painful nodule with intact mucosa was palpated on the left side of the vagina. Histological features were compatible with adenoid cystic carcinoma. Often, such lesion is clinically misdiagnosed as a cyst or inflammation. The present case was carried out with an impression of endometriosis. The possibility of cancer should be considered in any female older than 40 years of age with a lesion near the Bartholin’s glands.

## Introduction

Primary Bartholin’s gland cancer is rare, comprising 0.1 to 7% of vulvar carcinoma ^1^^-^^5^ and 0.001% of all malignancies in the female genital tract.^1^^-^^4^^,^^6^^,^^7^ Adenocarcinoma, squamous cell carcinoma, adenosquamous cell carcinoma, transitional cell carcinoma, and adenoid cystic carcinoma are various histological types of malignant tumors reported in the Bartholin’s gland. Adenoid cystic carcinoma (ACC) is rare, accounting for approximately 10-15% of all Bartholin’s gland tumors.^2^^-^^5^ ACC is a slow growing malignant tumor with a tendency for local recurrence and sometimes distance metastases. Signs and symptoms include pain, burning sensation and palpable mass.^2^^,^^3^^,^^5^ Only about 80 cases have been reported in English literature^6^ which often such lesion is clinically misdiagnosed as a cyst or inflammation. Herein, we report a case of ACC of Bartholin’s gland exhibiting vulvar pain, burning sensation and solid mass that clinically mimics endometriosis.

## Case Report

**Figure 1:**
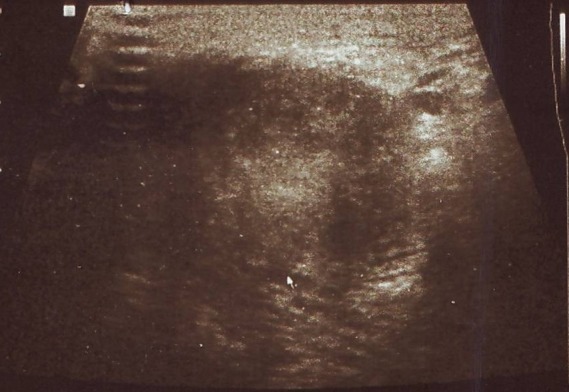
Vulva sonography shows hypoechoic mass 2.9×2.5×2.1 cm.

**Figure 2:**
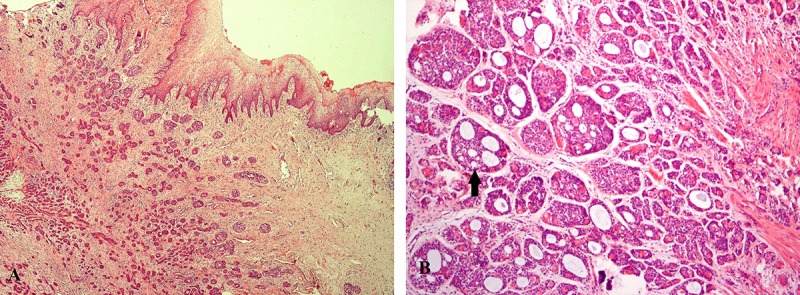
A) Vulvar mucosa is intact over the tumor (hematoxylin-eosin, magnification ×40). B) Cribriform pattern of tubules (arrow) and gland like elements (hematoxylin-eosin magnification ×100).

**Figure 3:**
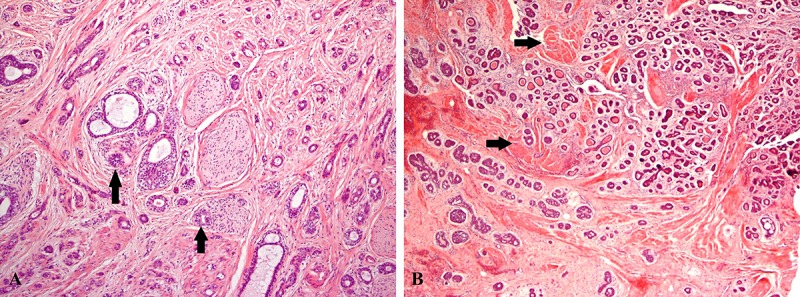
A) Perineural invasion was observed (arrow). B) The tumor had invaded to the adjacent muscle (arrows) (hematoxylin-eosin, magnification ×100).

A 42-year-old woman, G4L4 with regular menstruation was referred to the Gynecology Department of Hazrat Zeinab Hospital, which is affiliated with Shiraz University of Medical Sciences. The main complaint was a palpable painful mass and burning sensation on the left side of the vulva during the preceding two months. She did not have a vaginal discharge and her past medical history was ordinary except for a bilateral tubal ligation, which was performed 14 years earlier. Based on vaginal examination, a 3×2-solid fixed painful nodule was palpated that extended up to 2-3 cm above the hymenal ring on the left side of the vagina. The vaginal mucosa was intact and no groin lymphadenopathy was detected. Sonography revealed a solid hypoechoic mass measuring 2.9×2.5×2.1 cm on the left side of the vulva (figure 1). The patient’s laboratory data upon admission, including hematology, serum biochemistry were within normal limits. Consequently, the diagnosis of endometriosis was made. During the operation, a firm ill-defined mass with deep infiltration was detected in the anatomic region of Bartholin’s gland. This was extended to the vaginal wall and the pelvic floor. On gross examination, the excised mass was fragmented, creamy-brownish and solid measuring 5×4×4 cm. Microscopic examination revealed tubules with a cribriform pattern and gland like elements where few contained granular basophilic material and surrounded by hyaline stroma. The tumor cells were relatively uniform with scant cytoplasm and hyperchromatic round to the oval nuclei without nucleoli (figure 2A and 2B). Multifocal perineural invasion was observed and the tumor had invaded into the adjacent muscle (figure 3A and 3B). Histological features were compatible with ACC. Surgical margins were involved by the tumor and the overlying mucosa was intact. Although the transition from normal glandular epithelium to neoplastic epithelium was not identified, the carcinoma was considered to originate from the Bartholin’s gland. Subsequently, an examination under anesthesia showed no palpable residual tumor tissue. Abdominal and pelvic sonography, magnetic resonance imaging, thoracic radiography, and mammography showed no abnormality. The patient was referred for radiotherapy treatment and after 19-month, the patient was disease-free.

## Discussion

Adenoid cystic carcinoma was first recognized in 1853 as a variant of adenocarcinoma. It is most frequently seen in the salivary gland, breast, and skin.^5^ In the female genital tract, the cervix is the most common site of ACC but is rare in Bartholin’s gland.^5^^,^^7^ The diagnostic criteria for primary carcinoma of Bartholin’s glands are; i) the tumor is located in Bartholin’s gland area, ii) transition between normal epithelium to neoplastic epithelium is identified, and iii) no tumor is detected in other sites.^2^^,^^4^^,^^5^ Cases with transition from normal epithelium to neoplastic epithelium that are not identified, as in our case, are considered as primary carcinomas of Bartholin’s gland if the tumor completely replaces the gland and the overlying skin involvement or ulceration is absent.^1^

The classic histological pattern of ACC is a cribriform arrangement of tubules and gland-like elements, which are composed of nests and columns of uniform malignant cells separated by a hyaline stroma.^2^^,^^3^^,^^5^^,^^7^ Perineural invasion is a characteristic microscopic feature.^2^^,^^5^^,^^7^

The mean age at the time of diagnosis is 49 years with a range of 25-80 years.^2^^,^^6^^,^^8^^,^^9^ Signs and symptoms are nonspecific and composed of pain, burning sensation, palpable mass, dyspareunia or pruritus.^2^^,^^3^ Painful nodule is the most common symptom.^5^ Clinically, the painful swelling of the Bartholin’s gland area may be confused with Bartholin’s duct cyst and abscess treated by drainage or marsupialization which causes delay in diagnosis and treatment.^2^^,^^3^^,^^5^^,^^8^ In our case, the lesion was diagnosed as endometriosis. Consequently, the possibility of cancer should be considered in any women older than 40 years of age with a lesion near the Bartholin’s glands.^2^^,^^3^^,^^5^ Fine needle aspiration cytology is useful for a definite preoperative diagnosis. Tightly cohesive monomorphic cell clusters with magenta colored globule on the MGC stained smear and three-dimensional elements with a cribriform pattern are characteristic.^5^^,^^8^ ACC of Bartholin’s gland is a slow growing tumor but is locally invasive with characteristic perineural and lymphatic invasion that leads to local recurrence and the symptoms.^2^^,^^5^^,^^8^ Five- and a ten-year survival rate are 71% to 100% and 59% to 100% respectively.^6^ Distant metastasis via hematogenous spread may occur after a long disease-free period of time. The most common sites of metastasis are the bone and lung. As reported, it occurs less frequently as liver, kidney and brain metastasis.^7^^,^^9^ Primary treatment for this type of cancer is surgical removal. No clear consensus exists over an optimal surgical management protocol of Bartholin’s gland ACC due to its rarity and lack of a well-defined prognostic factor.^5^^,^^9^^,^^10^ The recommended surgical treatment is a simple or radical vulvectomy with or without lymph node dissection.^2^^,^^6^ Yang et al. showed a higher recurrence rate in patients undergoing simple excision compared with those undergoing radical vulvectomy (68.9% vs. 42.8%, respectively).^5^ It also showed the same recurrence rate in patients with negative margin (52.1%) and in patients with positive margin (52.9%). However, half of the patients with positive margins received adjuvant radiotherapy.^5^ Thus, it seems that surgical margin status is the most important factor for the determination of recurrence.^3^ Adjuvant radiotherapy is recommended when surgical margins are positive.^5^^,^^6^ The patient in this case report underwent simple mass excision with adjuvant radiotherapy due to positive surgical margins.

## Conclusion

Adenoid cystic carcinoma is a rare carcinoma of the vulva. Painful nodule is the most common symptom and the lesion is often overlooked as a benign process. Therefore, the possibility of cancer should be considered in any women older than 40 years of age with a mass near the Bartholin’s glands. 

## Consent

Written informed consent was obtained from the patient for the publication of this case report as well as accompanying images. A copy of the written consent is available for review by the Editor-in-Chief of this journal. 
